# Physicochemical properties, bond strength, and antibiofilm activity of a powder-gel bioceramic endodontic sealer

**DOI:** 10.1590/0103-644020246325

**Published:** 2025-12-01

**Authors:** Jéssica Arielli Pradelli, Pedro Luís Busto Rosim, Airton Oliveira Santos-Junior, Mario Tanomaru-Filho, Juliane Maria Guerreiro-Tanomaru

**Affiliations:** 1 Department of Restorative Dentistry, São Paulo State University(UNESP), School of Dentistry, Araraquara, SP, Brazil

**Keywords:** dental materials, endodontic sealers, bioceramics, physicochemical properties, antimicrobial

## Abstract

Physicochemical properties, bond strength, and antibiofilm activity of a powder-gel bioceramic endodontic sealer, Sealer Plus BC (SPBC, MK Life, Porto Alegre, RS, Brazil), were evaluated in comparison with Bio-C Sealer (BCS, Angelus, Londrina, PR, Brazil), a reference bioceramic sealer, and AH Plus Jet (AHP, Dentsply DeTrey, Konstanz, Germany), a standard resin-based sealer. The setting time, pH, solubility, flow, and radiopacity were evaluated in accordance with ISO 6876:2012 standards. Bond strength was measured using the Push-Out test. Antibiofilm activity against *Enterococcus faecalis* alone or in combination with *Candida albicans* was assessed using a modified direct contact test with the eluate of fresh sealer (25 mg/mL) and after 24 hours of material setting. The data were analyzed using ANOVA and Tukey statistical tests (α = 0.05). SPBC exhibited higher Setting Time values compared to BCS and lower values compared to AHP (p < 0.05). BCS and SPBC had higher solubility values than 3% (p<0.05). BCS demonstrated flow values higher than those of SPBC and AHP (p<0.05). SPBC showed lower radiopacity (p<0.05) and higher bond strength (p<0.05) than BCS and AHP. SPBC and BCS exhibited alkaline pH throughout all evaluated periods. They demonstrated antimicrobial activity against *Enterococcus faecalis* and/or *Candida albicans* in the eluate of fresh sealer and 24 hours after setting (p < 0.05). In conclusion, SPBC powder-gel sealer, as a filling material, meets most ISO 6876 standards for physicochemical properties, demonstrates bond strength to dentin, and exhibits antimicrobial activity against both dual and single-specimen biofilms. However, it has a solubility greater than 3%.

## Resumo

Este estudo avaliou as propriedades físico-químicas, resistência de união e a atividade antibiofilme de um novo cimento endodôntico biocerâmico pó-líquido Sealer Plus BC (SPBC, MK Life, Porto Alegre, RS, Brasil) comparado ao Bio-C Sealer Sealer (BCS, Angelus, Londrina, Paraná, Brasi) e AH Plus Jet (AHP, Dentsply DeTrey, Konstanz, Alemanha).Tempo de presa, pH solubilidade, escoamento e radiopacidade foram avaliados de acordo com normas ISO 6876:2012. Resistência de união foi avaliada por meio do teste de *Push-out*. Atividade antimicrobiana contra biofilme mono e dual-espécie de *Enterococcus faecalis* e *Candida albicans* foi investigada por teste de contato direto modificado com eluído do cimento fresco (50mg/ml) ou 24 horas após presa dos materiais. Os dados foram submetidos aos testes ANOVA e Tukey (α=0,05) SPBC apresentou tempo de presa maior que BCS e menor que AHP (p<0,05). BCS e SPBC apresentaram solubilidade maior que 3% (p<0,05). BCS apresentou maior escoamento que SPBC e AHP (p<0,05). SPBC apresentou menor radiopacidade (p<0,05) e maior resistência de união (p<0,05) que BCS e AHP. SPBC e BCS apresentaram pH alcalino em todos os períodos avaliados, bem como atividade antimicrobiana contra *E. faecalis* e/ou *C. albicans* por maio da avaliação do eluído do cimento fresco e após 24 horas de presa (p<0,05). Concluindo, o cimento pó-líquido SPBC como cimento endodôntico apresenta propriedades físico-químicas de acordo com os padrões ISO 6876, além de resistência de união à dentina e atividade antimicrobiana contra biofilme dual e mono espécime. No entanto, demonstrou solubilidade acima de 3%.

**Figure:**
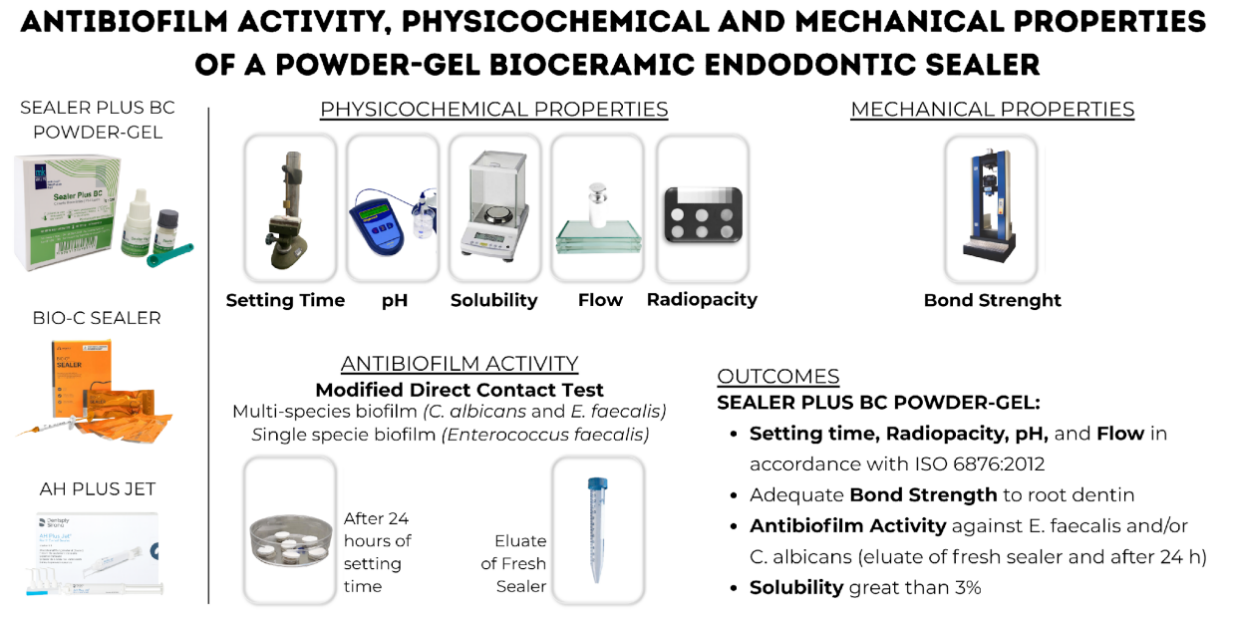

## Introduction

Endodontic sealers must possess adequate physicochemical and mechanical properties^1^ ^,^ ^2^, in addition to antimicrobial potential, which contributes to sealing and preventing reinfection^3^. Calcium silicate-based endodontic sealers, which belong to the broader category of bioceramic sealers, exhibit an alkaline pH^2^, flow, and radiopacity according to ISO 6876/2012 standards^2^ ^,^ ^4^. Furthermore, these sealers demonstrate bond strength to root dentin^2^ and antibiofilm activity against *Enterococcus faecalis* ^3^. However, bioceramic endodontic sealers, including those based on calcium silicate, tend to have solubility higher than that recommended by the ISO 6876 standards^1^.

Sealer Plus BC powder-gel (SPBC, MK Life, Porto Alegre, RS, Brazil) is a calcium silicate-based sealer composed of calcium silicates, calcium hydroxide, zirconium oxide, and a gel of propylene glycol and deionized water^5^. According to the manufacturer's recommendations, this material should be handled consistently for clinical applications as a repair material or endodontic sealer^5^. Bioceramic sealers in powder-gel composition undergo a hydration reaction upon manipulation due to the presence of water^6^. Additionally, they may have lower filling and flow capacities than ready-to-use bioceramic sealers, such as Bio-C Sealer^7^. This sealer is a biocompatible and bioactive, ready-to-use bioceramic sealer ^8^ ^,^ ^9^ presenting low porosity and voids^9^. It has an appropriate setting time, alkalization capacity, low volumetric change, flow, and radiopacity^10^. However, it exhibits higher solubility than recommended by the ISO 6876 standard^10^. AH Plus Jet (AHP, Dentsply, De-Trey, Konstanz, Germany) is an epoxy resin-based sealer considered the gold standard in terms of physicochemical properties. AHP has adequate setting time, radiopacity, flow, low dimensional change, and solubility values^1^ ^,^ ^4^ ^,^ ^10^.

Adequate bond strength between endodontic sealer and root dentin may be associated with a better interface, thus reducing microbial infiltration^11^. A powder-liquid bioceramic sealer demonstrated higher bond strength values than ready-to-use bioceramic sealers. This was likely related to the release of a larger quantity of calcium ions and more extensive biomineralization at the dentin-sealer interface^12^. In contrast, the bond strength of epoxy resin-based sealers is due to covalent bonds between the epoxy resin and collagen amino groups^12^.

Endodontic treatment failure is often associated with the persistence of infection^13^. *Enterococcus faecalis*, a gram-positive bacterium commonly present in the endodontic microbiota, has a high ability for dissemination in the root canal system and potential for survival in alkaline pH^13^. *Candida albicans,* a fungus, is known to contribute to endodontic therapy failure because of its ability to form robust biofilms and its resistance to conventional disinfection techniques^13^. The use of multi-species biofilm in the direct contact test reveals similarity to the clinical condition^14^, as *E. faecalis* demonstrates greater resistance when coexisting with *C. albicans* ^14^. There are no reports in the literature about the antimicrobial activity and bond strength of Sealer Plus BC powder-gel.

Therefore, this study aimed to evaluate the setting time, pH, radiopacity, flow, and solubility of Sealer Plus BC powder-gel bioceramic sealer. The bond strength and antibiofilm activity against *E. faecalis* and/or *C. albicans* were also evaluated. Bio-C Sealer, a pre-mixed bioceramic sealer, and AH Plus Jet, an epoxy resin-based sealer, were used as reference materials for comparison. The null hypothesis stated that there would be no differences among the tested sealers in terms of their properties, such as setting time, pH, radiopacity, flow, solubility, bond strength to root dentin, and antibiofilm activity against *E. faecalis* and/or C*. albicans*.

## Materials and methods

The endodontic sealers utilized in this study, along with their manufacturers, composition, and proportions, are detailed in Table 1. AH Plus Jet was manually mixed by combining equal parts of the two pastes, as shown in a previous study^15^. Bio-C Sealer and Sealer Plus BC were manipulated according to their respective manufacturers' instructions. Sealer Plus BC was mixed in a ratio of 7 scoops of powder to 5 drops of gel, as determined by a pilot study, to achieve optimal flowability as an endodontic sealer. In this study, Bio-C Sealer and AH Plus Jet were used as control groups to compare with Sealer Plus BC powder-gel. Bio-C Sealer is a bioceramic sealer, while AH Plus Jet is an epoxy resin-based sealer. These control groups were selected to assess the performance of Sealer Plus BC in comparison to established endodontic sealers.

**Table 1:** Material, manufacturers, composition, and proportion of the evaluated sealers

Material	Manufacturer	Composition	Proportion
Sealer Plus BC powder-gel (SPBC)	MK Life (Porto Alegre, RS, Brazil)	Powder: zirconium oxide, tricalcium silicate, dicalcium silicate, calcium hydroxide. Gel: propylene glycol and deionized water	7 scoops of powder: 5 drops of gel
Bio-C Sealer (BCS)	Angelus (Londrina, PR, Brazil)	Calcium silicates, calcium aluminate, calcium oxide, zirconium oxide, iron oxide, silicon dioxide, and a dispersing agent.	Ready to use
AH Plus Jet (AHP)	Dentsply (De-Trey, Konstanz, Germany)	Bisphenol A/F epoxy resin, calcium tungstate, zirconium oxide, silica, iron oxide pigments, dibenzyl diamine, amino adamantane, silicone oil.	1:1 (paste/paste)

### Sample Size Calculation

The sample size for each test (Setting Time, pH, Solubility, Flow, Radiopacity, Push-Out Bond Strength Evaluation, and Modified Direct Contact Test) was individually calculated using the G*Power software (version 3.1.7 for Windows, Heinrich Heine University of Düsseldorf, Germany), with an alpha error of 0.05 and a beta power of 0.90 for each test, based on previous studies ^10^ ^,^ ^16^ ^,^ ^17^. The minimum required sample size for each test was n = 6, ensuring statistical power to detect significant differences.

### Setting Time

Plaster molds that had been soaked in distilled water for 24 hours were used for the bioceramic sealers, while metal molds were used for the AHP, both with a diameter of 10 mm and a thickness of 1 mm, and filled with the sealers (n=6). The setting time (ST) was evaluated according to ISO-6876 standards^18^. A Gilmore needle, weighing 100 ± 0.5 g with an active tip diameter of 2 ± 0.1 mm, was employed, and the materials were incubated at 37°C with 95% humidity. The setting time was measured in minutes (min) as the time elapsed between the manipulation of the sealer when the Gilmore needle no longer left marks on the surface of the materials.

### pH

Polyethylene tubes measuring 10 x 1.6 mm (length x diameter) were filled with the sealers (n = 10) using a syringe or directly from the material's syringe, and then individually immersed in 10 mL of deionized water. The samples were incubated at 37°C, and pH measurements were taken at intervals of 1, 3, 7, 14, 21, and 28 days. Distilled water was used as the Control (C). The pH measurements were conducted in triplicate using a calibrated digital pH meter (Digimed Analítica Ltda., Grupo Digicrom Analítica, São Paulo, SP, Brazil).

### Solubility

The solubility of the materials was evaluated according to the ANSI/ADA standard, with adaptations by Carvalho-Junior et al.^19^. Sealer discs measuring 7.75 mm in diameter and 1.5 mm in height were prepared, with a nylon thread (n = 6) embedded in the sealer mass. After 24 hours in an oven at 37 °C, the specimens were weighed using a calibrated precision balance (Adventurer AR2140; Ohaus Corporation, Parsippany, NJ) to determine their initial mass. The nylon thread was used to suspend the sealer discs in a plastic container containing 7.5 mL of distilled water at 37ºC, for 7 days, without allowing any contact between the sealer and the inner surface of the container. The discs were removed from the distilled water, dried with absorbent paper, and inserted into a dehumidifier until the mass stabilized. Solubility was determined by calculating the mass loss after immersion, which was expressed as a percentage (%).

### Flow

The flow test was conducted in accordance with the ISO 6876:2012 standard^18^. A volume of 0.05 ± 0.005mL of the sealer (n = 6) was applied to a glass plate. A new glass plate weighing 20 g was placed on top of the sealer, and a load of 100 g was applied on the central region of the plate. After 7 minutes, the maximum and minimum diameters of the material flow were measured using a digital caliper in millimeters (mm). If the difference between the diameters was less than 1 mm, the average value was recorded.

### Radiopacity

Four test specimens, one of each sealer, measuring 10 mm in diameter x 1 mm thick, were placed on occlusal radiographic films (Insight-Kodak Comp, Rochester, NY) and exposed next to an aluminum scale with variable thickness (2 to 16 mm, in increments of 2 mm). An X-ray apparatus (Instrumentarium Dental, Tuusula, Finland) operating at 60 kV, 7 mA, and 0.32 pulses per second, with a focus-film distance of 33 cm, was used. The films were developed in a conventional automatic processor (Dent-X 9000; Dent-X, Elmsford, NY). After this, the radiographs were digitized, and the images were transferred to Image Tool 3.0 software (University of Texas Health Science Center at San Antonio, San Antonio, TX). To evaluate the radiopacity of the materials, the areas corresponding to each degree on the aluminum scale and the areas of the sealers were selected. This radiopacity was expressed in terms of the equivalent aluminum thickness (in mm/Al).

### Push-Out Bond Strength Evaluation

Bovine maxillary incisors were selected, and their crowns and apical 2 mm were removed. The roots were then embedded in lamination resin (Maxi Rubber, São Paulo, SP, Brazil) and transversely cut into 2 mm thick slices using an Isomet 1000 (Instmed, São Paulo, Brazil). Root canal preparation was standardized using a B2 parallelometer (Bio-Art, São Carlos, SP, Brazil) and a 702 tapered carbide bur (Labor dental, São Paulo, SP, Brazil) with 1% sodium hypochlorite (NaOCl) irrigation. After preparation, the canals were irrigated with 17% EDTA for 3 minutes, rinsed with 5 mL of water, and then filled with sealers (n = 10). After incubating for 7 days at 37ºC and 95% humidity, a *Push-out* test was conducted on an Emic DL 2000 machine (Instrom, São José dos Pinhais, PR, Brazil) with a load cell of 1 kN operating at a constant speed of 0.5 mm/min and a cylindrical stainless-steel punch with a diameter of 1.2 mm (approximately 70% of the canal diameter)^20^ ^)^ was until the material was displaced. The maximum load was recorded in Mega Pascal (MPa) after entering the height and inner radius of the specimen into the universal testing machine.

### Evaluation of Antibiofilm Activity (Modified Direct Contact Test)

All experimental procedures were performed inside a previously sterilized laminar flow hood to prevent contamination. Multi-species biofilms comprising *Enterococcus faecalis* and *Candida albicans*, as well as single-species biofilms of *E. faecalis*, were cultured from standard strains (*E. faecalis* ATCC 29212 and *C. albicans* ATCC 10231). The biofilms were cultivated on hydroxyapatite discs (5 mm diameter, 1 mm thick) for 5 days for *E. faecalis* and 7 days for *C. albicans* ^21^ ^,^ ^22^. To ensure aseptic conditions, the hydroxyapatite discs were sterilized before use, while the sealer discs were exposed to UV light for 30 minutes. Additionally, the distilled water used for the eluate was previously sterilized, as were the culture media. Direct contact tests were conducted using two forms of the sealer: the eluate of fresh sealer (25 mg/mL) and sealer discs after 24 hours of setting under humid conditions. Each test group consisted of six replicates (n = 6). Distilled water served as the control for the eluate, while Teflon discs were used as controls for the sealer discs (C). After 15 hours of contact, the hydroxyapatite discs were subjected to serial dilution, and triplicate plating was performed on selective culture media: Tryptic Soy Agar (TSA) for *E. faecalis* biofilms and on M-*Enterococcus* Agar Base and Sabouraud Dextrose Chloramphenicol Agar (SDA) for the multi-species biofilms. The plates were incubated at 37°C for 48 hours, after which colony-forming units (CFU/mL) were counted. Data were log-transformed (log10) for statistical analysis.

### Statistical analysis

All data were first assessed for normality using the Shapiro-Wilk test and found to meet the assumptions for parametric testing. The physicochemical, mechanical, and antibiofilm data were analyzed using one-way ANOVA to compare groups, followed by post-hoc Tukey tests. A significance level of α = 0.05 was used for all statistical analyses.

## Results

The physicochemical and mechanical properties data are represented in Table 2.

**Table 2:** Mean and ± standard deviation of setting time, solubility, flow, radiopacity, and bond strength of the evaluated sealers.

Test	Sealer Plus BC	Bio-C Sealer	AH Plus
Setting time (min)	356.17(±21.38)^b^	202.33(±12.86)^c^	1252.67(9.46)^a^
Solubility (%)	15.13(±1.16)^a^	4.71(±0.80)^b^	0.03(±0.06)^c^
Flow (mm)	23.70(±1.29)^b^	31.45(±1.22)^a^	21.65(±0.89)^c^
Radiopacity (mm/Al)	3.97(±0.51)^c^	5.57(±0.35)^b^	15.64(±0.65)^a^
Bond Strength (MPa)	4.39(±0.79)^a^	1.57(±0.63)^b^	1.84(±0.42)^b^

Different letters on the same line indicate statistically significant differences (p<0.05).

SPBC had a longer setting time than BCS but shorter than AHP (p<0.05). SPBC had the highest solubility, followed by BCS, while AHP had the lowest (p<0.05). SPBC had a lower flow rate than BCS but higher than AHP (p<0.05). SPBC had the lowest radiopacity, while BCS showed intermediate values, and AHP had the highest (p<0.05). SPBC had the highest bond strength (p<0.05), while BCS and AHP had similar bond strength (p>0.05).

Table 3 displays the pH values of the tested sealers. On day 1, BCS had the highest pH, followed by SPBC and AHP (p<0.05). From days 3 to 28, SPBC and BCS had similar pH values, both higher than AHP (p<0.05). At 21 days, SPBC had the highest pH, followed by BCS and AHP (p<0.05). The control group consistently had the lowest pH values (p<0.05), with AHP and the control group showing similar values at 14 days (p>0.05).

**Table 3:** Mean and ± standard deviation of pH of the sealers evaluated after time intervals of 1, 3, 7, 14, 21, and 28 days.

Period	Sealer Plus BC	Bio-C Sealer	AH Plus	Control
1 day	9.63(±0.35)^b^	10.79(±0.44)^a^	9.01(±0.53)^c^	7.40(±0.17)^d^
3 days	9.28(±0.20)^a^	9.67(±0.70)^a^	8.18(±0.55)^b^	7.57(±0.12)^c^
7 days	8.86(±0.35)^a^	9.38(±0.54)^a^	7.92(±0.41)^b^	7.59(±0.23)^c^
14 days	9.63(±0.48)^a^	9.80(±0.66)^a^	7.96(±0.41)^b^	7.70(±0.17)^b^
21 days	10.21(±0.20)^a^	9.69(±0.56)^b^	7.96(±0.41)^c^	7.42(±0.14)^d^
28 days	10.02(±0.25)^a^	9.78(±0.47)^a^	7.95(±0.42)^b^	7.46(±0.18)^c^

Different letters on the same line indicate statistically significant differences (p<0.05).

The antibiofilm activity results of the sealers are represented in Table 4. SPBC and BCS demonstrated superior antibiofilm activity in all tests (p<0.05). AHP exhibited higher antibiofilm activity than the control (p<0.05), except in tests using the eluate of fresh sealer against *Enterococcus faecalis* and *Candida albicans* (p>0.05).

**Table 4:** Mean ± Standard Deviation of the antimicrobial activity of the evaluated materials, expressed in Colony Forming Units (CFU), as determined by the modified direct contact test.

		Sealer Plus BC	Bio-C Sealer	AH Plus	Control
Eluate of fresh sealer	*E. faecalis*	0.00(±0.0)^c^	0.00(±0.0)^c^	4.77(±2.23)^b^	6.58(±0.22)^a^
	*E. faecalis* + *C. albicans*	0.00(±0.0)^b^	0.00(±0.0)^b^	6.41(±0.50)^a^	6.55(±0.21)^a^
Sealer 24 hours after setting	*E. faecalis*	0.00(±0.0)^c^	0.00(±0.0)^c^	5.28(±0.50)^b^	6.83(±0.11)^a^
	*E. faecalis* + *C. albicans*	0.00(±0.0)^c^	0.00(±0.0)^c^	5.40(±0.33)^b^	7.60(±0.23)^a^

Different letters on the same line indicate statistically significant differences (p<0.05).

## Discussion

The physicochemical properties, bond strength, and antibiofilm activity of the new powder-gel sealer, Sealer Plus BC (SPBC), were compared to the ready-to-use bioceramic sealer Bio-C Sealer (BCS) and the epoxy resin-based AH Plus Jet. The null hypothesis was rejected as the materials showed differences in the evaluated properties.

SPBC exhibited longer setting time than BCS but less time than AHP. The setting time for the materials evaluated was in accordance with that recommended by the ISO 6876 specification (between 30 minutes and 72 hours) ^18^. The higher water content in hydraulic sealers can be related to extended setting time^23^. The SPBC can be used as a repair material or an endodontic sealer, depending on the powder-to-gel ratio^5^. The manufacturer does not provide a specific ratio, and there are no studies on the ideal ratio for each use^5^. In this study, a powder-liquid ratio of 7:5 was selected for SPBC to enhance its flow as a root canal sealer.

Endodontic sealers must have adequate flow to promote effective root canal filling.^4^. All the sealers in this study exhibited flow in accordance with ISO 6876 standards (≥ 17mm).^18^. The lower flow of SPBC compared to BCS can be related to the powder-gel composition in relation to ready-to-use sealers^7^. Similar flow values have been demonstrated for BCS and AHP^4^. In this study, the powder-gel ratio for SPBC was based on a previous pilot evaluation of its ideal proportion for proper flow as a filling sealer. However, increasing the liquid content in the hydraulic sealer can alter physicochemical properties and radiopacity^6^. Bioceramic sealers with higher water content may result in longer setting times, higher pH levels, increased calcium ion release, and volumetric changes^23^.

The radiopacity of the filling material enables the assessment of the filling of the root canal in relation to the surrounding structures^4^. In this study, SPBC demonstrated lower radiopacity than BCS and AHP; however, all sealers met the ISO 6876 requirements (>3 mm Al)^18^. SPBC, BCS, and AHP contain zirconium oxide as the radiopacifying agent^4^ ^,^ ^5^ ^,^ ^24^. Adding 30% zirconium oxide to calcium silicate sealers ensures sufficient radiopacity without impacting hydration, pH, or antimicrobial activity^25^. AH Plus sealer includes calcium tungstate as an additional radiopacifier, enhancing its radiopacity^4^ ^,^ ^24^.

In this study, SPBC showed higher bond strength compared to BCS and AHP. Bioceramic sealers have the capacity for mineral deposition in intratubular dentin, thereby establishing micromechanical and chemical interaction with the walls of the root canal^26^. Powder-liquid bioceramic sealers have previously been demonstrated to have higher bond strength than ready-to-use bioceramic sealers^12^. BioRoot RCS and ProRoot ES are tricalcium silicate-based powder-liquid sealers, like SPBC, that exhibit higher bond strength values compared to the ready-to-use bioceramic sealer EndoSequence BC^12^. Additionally, the powder-liquid bioceramic sealer exhibited higher bond strength than the ready-to-use calcium silicate-based sealers^27^. The higher bond strength of powder-liquid sealers may be attributed to their increased release of calcium ions and apatite precipitation, thereby enhancing their biomineralization capacity^12^ ^,^ ^26^.

The study evaluated solubility according to ISO 6876:2002, with adjustments to sample dimensions as described by Carvalho-Junior et al.^19^. The standard recommends a solubility level below 3% after 24 hours of immersion in distilled water^18^. AHP exhibited the lowest solubility, while SPBC and BCS exceeded the 3% threshold. Ready-to-use bioceramic sealers have shown solubility above 3% in previous studies ^4^ ^,^ ^10^. The high solubility in ISO tests is attributed to the hydrophilic property of calcium silicate sealers and the dehydration process for mass loss measurement^1^ ^,^ ^4^ ^,^ ^10^. However, micro-CT tests show minimal volumetric change for these sealers, providing a more accurate representation of volumetric behavior^10^. This highlights the importance of using additional methods to assess the clinical effectiveness of bioceramic sealers. AHP, an epoxy resin-based sealer, forms stable covalent bonds with a highly stable polymer^28^.

The solubility of bioceramic materials can be attributed to their alkalinizing capacity^29^. In this study, the bioceramic sealers SPBC and BCS exhibited higher pH values than AHP across all evaluated time intervals. Epoxy resin-based sealers, such as AHP, exhibit antimicrobial activity during the setting reaction through the release of amines that disrupt bacterial membranes.^30^. Calcium silicate-based sealers release calcium and hydroxyl ions during setting, increasing environmental alkalinity^27^, favoring biocompatibility^11^ and antimicrobial activity^3^.

The modified direct contact test (MDCT) was used to assess the antibiofilm activity of materials in contact with microbial biofilms^4^. The 15-hour test simulates prolonged exposure of biofilms to endodontic sealers, allowing for assessment of sustained ion release, pH increase, and antimicrobial effectiveness ^10^ ^,^ ^31^. *E. faecalis* and *C. albicans* were chosen for their involvement in persistent endodontic infections and resistance in biofilms^10^. The use of multi-species biofilms in the test reflects clinical conditions^13^ ^,^ ^14^ ^,^ ^30^. Previous studies on bioceramic sealers have also used similar methodologies, supporting the validity of this approach^10^. Hydroxyapatite discs were selected as the substrate for biofilm growth due to their similarity to dentin in terms of mineral composition, surface roughness, and ability to support microbial adhesion. This makes them a suitable model for assessing the antimicrobial effectiveness of endodontic sealers^32^ ^,^ ^33^ ^,^ ^34^.

The antibiofilm activity revealed that SPBC and BCS exhibit antimicrobial potential against *E. faecalis* and/or *C. albicans* in the eluate of fresh sealer and after 24 hours of setting. In agreement, other studies have also reported antimicrobial activity of bioceramic endodontic sealers against *E. faecalis* and/or *C. albicans* ^3^ ^,^ ^4^ ^,^ ^10^. The antibiofilm properties of bioceramic sealers may be attributed to the release of calcium and hydroxyl ions, increasing the alkalinity of the environment^3^. An alkaline pH can disrupt the microbial cell structure, leading to DNA degradaton and protein damage, thereby enhancing the antimicrobial properties of bioceramic materials^3^ ^,^ ^35^.

The results of the present study had limitations in terms of direct clinical applicability. The assessment of setting time and solubility of Calcium silicate-based endodontic sealers has limitations due to their hydraulic characteristics^1^ ^,^ ^9^. Despite this, the findings enhance our understanding of the properties of the bioceramic materials studied^4^ ^,^ ^8^ ^,^ ^9^ ^,^ ^10^. Biofilm formation was assessed over 7 days to evaluate the initial antimicrobial activity. A shorter incubation period was chosen to test the sealer's effectiveness in biofilms of *E. faecalis* and/or *C. albicans*. However, more extended maturation periods should be considered in future research to simulate clinical conditions more accurately.

There are no parameters for comparison of the new Sealer Plus BC powder-gel sealer. Further research is needed to determine its suitability for clinical use. According to the methodology used and the results obtained, Sealer Plus BC powder-gel sealer shows setting time, radiopacity, pH, and flow in accordance with ISO 6876-2012 standards. It has adequate bond strength to root dentin and antibiofilm activity against *E. faecalis* and/or *C. albicans*, both in the condition of using the eluate of fresh sealer and that collected after 24 hours of setting. However, Sealer Plus BC has higher solubility than recommended by ISO 6876.
